# Ethnic and Gender Disparities in Healthy Ageing among People 50 Years and Older in South Africa

**DOI:** 10.3390/geriatrics6030079

**Published:** 2021-08-12

**Authors:** Supa Pengpid, Karl Peltzer

**Affiliations:** 1 ASEAN Institute for Health Development, Mahidol University, Salaya, Phutthamonthon, Nakhon Pathom 73170, Thailand; supaprom@yahoo.com; 2 Department of Research Administration and Development, University of Limpopo, Sovenga 0727, South Africa; 3 Department of Psychology, University of the Free State, Bloemfontein 9300, South Africa; 4 Department of Psychology, College of Medical and Health Science, Asia University, Taichung 41354, Taiwan; 5 Department of Human and Social Capabilities, Human Sciences Research Council, Pretoria 0001, South Africa

**Keywords:** healthy ageing, older adults, South Africa

## Abstract

Objective: this study aimed to determine the prevalence and correlates of healthy ageing in older adults living in the community in South Africa. Methods: the cross-sectional sample consisted of 3734 individuals (≥50 years) from the cross-sectional South African National Health and Nutrition Survey (SANHANES-1) in 2011–2012. Healthy ageing was assessed using a multidimensional concept, which includes five components: (1) absence of major illness, (2) absence of disability, (3) good mental health, (4) social engagement and (5) well-being or good health. Results: in general, 36.6% had a healthy ageing, including 73.3% had no major diseases, 87.1% were free of disability, 62.3% had good mental health, 73.0% were socially engaged and 64.0% had a high well-being. In the adjusted logistic regression analysis, male sex (Adjusted Odds Ratio-AOR: 1.33, 95% confidence interval-CI: 1.03–1.72), white population group (AOR: 3.46, 95% CI: 2.29–5.22) and coloured population group (AOR: 1.82, 95% CI: 1.34–2.47), were positively associated with healthy ageing, while increasing age (AOR: 0.96, 95% CI: 0.94–0.97), daily tobacco use (AOR: 0.56, 95% CI: 0.42–0.74), perceived underweight (AOR: 0.48, 95% CI: 0.34–0.66) and perceived overweight (AOR: 0.53, 95% CI: 0.34–0.81) were negatively associated with associated with healthy ageing. Conclusion: almost two in five older adults in South Africa were successfully ageing. Factors associated with healthy ageing included, younger age, male sex, population group (Whites, Coloureds), not daily tobacco users, not having underweight and overweight.

## 1. Introduction

In most African societies, due to rapid ageing, urbanization, changes in socioeconomic and cultural patterns and weakening of kinship support, older adults may receive less support and respect, becoming increasingly a burden on families and dependents, and healthy ageing becomes critical [1]. Over period of 20 years from 2002 to 2022, the projected increase of older adults (≥60 years) in South Africa is 69.9%, 5.7 million (9.1% of the population) by 2022 [2]. According to the World Health Organization (WHO) [3], healthy ageing is “the process of developing and maintaining the functional ability that enables wellbeing” and optimizing functional ability (“ability to meet one’s basic needs; ability to learn, grow and make decisions; mobility; ability to build and maintain relationships; and ability to contribute”) is a key to healthy ageing. Definitions of healthy ageing include “survival to a specific age, being free of chronic diseases, autonomy in activities of daily living, well-being, good quality of life, high social participation, only mild cognitive or functional impairment, and little or no disability” [4]. WHO [3] propagates that “governments and other stakeholders must invest in data to monitor healthy ageing across the life course”.

Studies on healthy ageing among older adults have been conducted largely in high-income countries. Taking into account the differences in socioeconomic contexts, culture, retirement and leisure in low-resource countries, such as South Africa, understanding healthy ageing among older adults in South Africa is important to provide the solutions needed to ‘promote healthy ageing, provide quality health and social care to the rapidly growing number of older adults in South Africa and address the imminent burden of health and social welfare of ageing in resource-poor settings.’ [5] (p. 178). One means to be used for evaluating the health status of older adults in South Africa is by assessing and monitoring healthy ageing (HA). The biomedical model of healthy ageing (BMHA) may include five components: “no major disease, no cognitive impairment, high physical functioning, no disability and active engagement with life” [6,7], and a multidimensional concept of healthy ageing (MMHA) may include five components, such as no major disease, no disability, good mental health, social engagement and well-being [8].

Using the MMHA, the prevalence of HA was among older adults (≥65 years) 18.6% in China [8], and in 15 European countries (≥50 years) 23.5% [9]. Using the BMHA, the prevalence of HA was among older adults (≥60 years) in China 13.2% [10], and in Singapore (≥60 years) 25.4% [7]. The proportion of five components of MMHA was, for example in China (≥65 years) “no major illness 75.1%, no disability 86.0%, no depression symptom 75.2%, active social/productive engagement 51.2% and life satisfaction 57.1%” [8], and the prevalence of five components of BMHA was, for example in China (≥60 years) no major diseases 41.7%, no disability 92.1%, high cognitive functioning 54.2%, high physical functioning 70.2% and active engagement with life 46.0% [10]. To our knowledge, we could not find any study on HA among older adults in South Africa, which led to this study.

Sociodemographic factors associated with HA may include younger age [8,11,12], male sex [8,11,12], higher income/wealth [8,12,13,14,15], ethnicity [7] and urban residence [16]. In addition, various health behaviours associated with HA have been found, including not smoking [17], alcohol consumption [8], physical activity [12,17,18,19], healthy diet [20] and a normal body mass index [17]. Studies on HA among older adults have been conducted primarily in high-income countries, except China. Taking into account the differences in socioeconomic contexts, culture, retirement and leisure in low-resourced countries in Africa, an understanding of HA among older adults in South Africa is important. Sociodemographic factors and health behaviour were hypothesized to influence HA in South Africa. Therefore, this study aimed to determine HA in older community-dwelling adults in South Africa.

## 2. Methods

### 2.1. Sample and Procedures

We analyse a subsample (50 years and older) that participated in the cross-sectional South African National Health and Nutrition Survey (SANHANES-1) in 2011–2012. Further details are provided elsewhere [21]. Briefly, “500 enumeration areas (EAs) representative of the sociodemographic profile (stratified by province, locality type and race or population group) of South Africa were identified, and a random sample of 20 households was selected from each EA.” [21]. All persons residing in the selected households were eligible to participate in an interview-administered questionnaire and health examination [21]. We restricted our sample to those that had complete HA measurements and were 50 years and older. Participants provided informed written consent, and the study protocol was approved by the Research Ethics Committee (REC) of the Human Sciences Research Council (REC 6/16/11/11).

### 2.2. Measures

#### Healthy Ageing

HA was assessed using a MMHA, including five components: (1) absence of major illness, (2) free from disability, (3) good mental health, (4) social participation and (5) life satisfaction/good health [8].

Absence of major illnesses were sourced from the questions, “Has a doctor or nurse or health worker at a clinic or hospital told you that you have or have had any of the following conditions…stroke, heart attack or angina, heart disease, diabetes, heart failure, rheumatic heart disease.” (Yes, No) [21].

Free of disability was measured based on ‘Activities of daily living (ADL)’ 6 items: (1) walking a long distance, (2) bathing or washing the whole body, (3) dressing, (4) moving through a room, (5) eating food and (6) getting to and using the toilet’. Responses ranged from 1 = no difficulty to 5 = extreme difficulty and were dichotomised into “no disability” (no difficulty and mild difficulty = 1) and “with disability” (moderate, severe and extreme difficulty = 0). Free of disability was defined as scoring 1 on all six items [22] (Cronbach’s alpha 0.87 in this sample).

Good mental health was evaluated with the Kessler psychological stress scale (K10) [23], which in a validation study in South Africa found that the ‘optimum cut-off of 16 produced 70% sensitivity and 67% specificity’ [24] and was defined as <16 scores on the K10. Cronbach’s alpha for K10 was 0.93 in this sample.

Social engagement was defined as not having difficulty in “Joining in community activities (for example, festivities, religious or other activities) in the same way as anyone else can” [21].

High well-being or overall health was assessed from the question, “In general, how would you rate your health today?” and defined as good or very good = 1 and moderate, bad, very bad = 0 [21].

HA was further assessed using BMSA, including five components: (1) absence of major illness (stroke, heart attack or angina, heart disease, diabetes, heart failure, rheumatic heart disease and no psychological distress), (2) free of disability (0 difficulty with ADL), (3) no cognitive impairment, (4) high physical function and (5) social participation.

No cognitive impairment was based on two questions on subjective cognitive complaints (a) Overall, in the last 30 days, how much difficulty did you have in concentrating or remembering things? and (b) In the last 30 days, how much difficulty did you have in learning a new task (e.g., learning how to acquire to a new place, learning a new game, learning a new recipe, etc.)? [25]. Responses ranged from 1 = none to 5 extreme/cannot do. No cognitive impairment was defined as none, mild or moderate difficulty vs. severe or extreme/cannot do on the two questions.

High physical function was defined as none or mild difficulty versus moderate, severe or extreme/can’t do any in both moving around and in “vigorous activities (‘vigorous activities’ require strenuous physical effort and cause large increases in breathing or heart rate)” [21].

### 2.3. Covariates and Confounders

Sociodemographic variables consisted of age, sex, population group and residential status.

Daily tobacco use was sourced from items on tobacco smoking and use of other tobacco [21].

Problem drinking was assessed with the 3-item “Alcohol Use Disorder Identification Test–Consumption (AUDIT-C)”. Total scores of the AUDIT-C ranged from 0 to 12. Scores of three or more in women and four or more in men indicate hazardous drinking [26]. The Cronbach alpha for the AUDIT-C in this sample was 0.89.

Physical activity was assessed with the “General Physical Activity Questionnaire (GPAQ)” [27,28], resulting into low, moderate and high physical activity.

Sedentary behaviour was assessed with two items, “on the time spent sitting or reclining (lying) on a usual weekday or weekend day (excluding sleeping)” [29] and categorized into 0 < 8 h, and 1 = 8 or more hours a day [30].

Fruit consumption “How many fruits do you usually eat per day?” Vegetable consumption “How many portions of vegetables, excluding potatoes, do you usually eat per day?” [21].

Perceived body weight was measured with the question, “Do you think you are underweight, normal weight or overweight?” [21].

### 2.4. Data Analysis

Descriptive statistics were applied to describe sociodemographic information, health indicators, and HA. Unadjusted and adjusted logistic regression was used to assess associations between sociodemographic, health risk behaviour and MMSA and BMSA. *p* < 0.05 was accepted as significant, missing values were excluded, and no multi-collinearity was found. Statistical analyses were conducted using “STATA software version 15.0 (Stata Corporation, College Station, TX, USA)”, taking the complex study design into account.

## 3. Results

### 3.1. Sample Characteristics

The total sample with complete HA measurements included 3734 people 50 years and older (median age = 59 years, interquartile range = 54–68), 56.8% were female and 43.2% were male. Majority (61.3%) of the participants were urban dwellers, and 60.3% belonged to the Black African population group. Almost two in five participants (18.7%) were currently using tobacco daily, 17.9% in problem drinking, 51.5% had low physical activity and 15.6% engaged in sedentary behaviour. Almost half of the participants (44.9%) had less than once a day fruits, 44.2% had less once a day vegetables, 11.0% perceived themselves to be underweight or 17.9% overweight. Overall, 36.6% had HA, including, 73.3% had no major diseases, 87.1% were free from disability, 62.3% had good mental health, 73.0% were socially engaged and 64.0% had high well-being (see Table 1).

Table 2 provides an overview of the prevalence of each HA component stratified by HA models and by age groups. In both models, the prevalence of HA decreased with age. Looking at the different HA components, all components declined with age (no disease, no disability, no cognitive impairment functioning, high physical function, social engagement, good mental health and well-being. Using the MMHA, 73.3% had no disease, 87.1% no disability, 62.3% good mental health and 64.0% well-being or good health, and using the BMHA, 49.7% had no disease, 87.1% no disability, 91.0% no cognitive impairment, 73.4% high physical functioning and 73.0% social engagement (see Table 2).

### 3.2. Associations with Healthy Ageing

In adjusted logistic regression analysis, male sex (Adjusted Odds Ratio-AOR: 1.33, 95% confidence interval-CI: 1.03–1.72), white population group (AOR: 3.46, 95% CI: 2.29–5.22) and coloured population group (AOR: 1.82, 95% CI: 1.34–2.47), were positively associated with MMHA, while increasing age (AOR: 0.96, 95% CI: 0.94–0.97), daily tobacco use (AOR: 0.56, 95% CI: 0.42–0.74), perceived underweight (AOR: 0.48, 95% CI: 0.34–0.66) and perceived overweight (AOR: 0.53, 95% CI: 0.34–0.81) were negatively associated with associated with MMHA. The correlates for BMHA were the same as MMSA. In addition, in unadjusted analysis, urban residence was associated with MMHA (see Table 3).

## 4. Discussion

To our knowledge, this study is the first to assess the prevalence and factors associated with HA among older adults (≥50 years) in a national community-based sample in South Africa. Using the MMHA, we found that more than one in three older adults (36.6%) in South Africa were successfully ageing, which is higher than in 15 European countries (≥50 years) 23.5% [9]. Using BMHA, we found that two in five older adults (40.3%) in South Africa were successfully ageing, which is higher than in China (13.2%, ≥60 years) [10]. Comparing the assessment of HA with MMHA and BMHA, this study found in contrast to previous research [8,9] that the rates of BMHA were higher than MMHA. The more flexible MMHA may be more useful for targeting identified deficiencies in public health interventions [9].

Using the MMHA, 73.3% had no disease, 87.1% no disability, 62.3% had good mental health, 73.0% social engagement and 64.0 well-being or good health, which compares to a study in China (≥65 years), as follows, no major illness 75.1%, no disability 86.0%, no depression symptom 75.2%, active social/productive engagement 51.2% and life satisfaction 57.1% [8]. Good mental health and social engagement were higher in this study than in the China study, which may be attributed to different modes of measurement and the South African sample being younger than the China sample [8].

Using BMHA, we found in this study that 49.7% had no disease, 87.1 no disability, 73.0% social participation, 73.4% high physical functioning and 91.0% no cognitive impairment, compared to a study in China (≥60 years) of active participation in life 46.0%, high physical function 70.2%, high cognitive functioning 54.2%, no disability 92.1% and no major diseases 41.7% [10]. The proportion of older adults with no cognitive impairment and social engagement were higher in this study than in the China (≥60 years) study [10], which may be attributed to different modes of measurement and the South African sample being younger than the China sample. Analysing the different components of HA by age groups, we found that the decline with age was significant for all HA components, which largely concurs with previous research [12].

We found that the younger age, male sex, population group (whites, coloureds), not daily tobacco users, not overweight and overweight were associated with MMHA and/or BMBA. Consistent with previous research [8,11,12], male sex was found to be associated with HA, which may be related to gender paradox in health (women living with worse health longer than men) [8]. These gender differences seem to be mainly attributed to men having higher no disability and social engagement than women. The found gender differences are consistent with research showing lower functional health among older women than among men in South Africa [31]. In addition, younger age was associated with HA in this study, which concurs with previous findings [8,11,12]. The negative association between age and HA is expected due to biological, functional and cognitive decline with age [7].

In line with some previous research [14], this study showed in an unadjusted analysis that urban residence was positively associated with HA. Urban residence may be associated with a higher educational and economic status and better access to health care, all of which could increase HA [16]. Good mental health, no disability, social engagement and well-being or good health were lower among older adults residing in rural compared to urban areas in this study. Social participation and mental health promotion should be promoted among older adults in rural areas in South Africa. Furthermore, programmes that involve more direct contact with older people, such as storytelling and personal ageing narratives, would allow older adults to be active subjects in research on health care and social policy services. This would promote a more inclusive model of HA in critic to active ageing policies and norms, which often limit those older individuals who are already excluded, discriminated and marginalised [32].

Furthermore, we found ethnic differences in the prevalence of HA, as found in previous research [7,10]. Compared to Black Africans, Whites and Coloureds were likely to have a higher prevalence of HA in this study. This result may largely reflect the life expectancy at birth by population group in South Africa, Whites and Coloureds have a higher life expectancy than Black Africans [33], and other racial or ethnic health disparities among older adults in South Africa [34].

Consistent with previous studies [8,17], we found that non-daily tobacco use and normal BMI were associated with HA. Based on longitudinal studies, consistent evidence was found that tobacco use is negatively associated with HA [35]. Smoking has been known to be associated with contributing to morbidity and mortality [35], and tobacco use cessation should be initiated in this older adult population in South Africa. Overweight/obesity has been shown to lead to chronic physical conditions [36]. Underweight has been found associated with poorer health outcomes [37], and low BMI was associated with non-survival [38]. However, in contrast with previous research [8,12,17,18,19,20], we did not find an association between alcohol use, physical activity, dietary behaviour and HA.

Study limitations include the cross-sectional design, the assessment of some variables by self-report. A bias may be less for diagnosed chronic conditions than for self-reported health. Some variables, such as mental or cognitive activity and socioeconomic status [8,11,12,13], that have been shown to influence HA were not assessed and should be included in future research. The assessment of cognitive functioning used only two questions, and future studies may use the Mini-Mental State Exam (MMSE) for general cognitive evaluation. The statistical models were adjusted for various confounding variables, but the findings may still have been confounded by other variables, such as psychological coping resources, not included in the analyses. Furthermore, the study focused on older adults living in the community and excluded institutionalised persons.

## 5. Conclusions

Almost two in five older adults in South Africa were successfully ageing. Factors associated with successful HA included, younger age, male sex, population group (Whites, Coloureds), not daily tobacco users, not having underweight and overweight. Cessation of tobacco use and reaching and maintaining normal body weight are major intervention targets to improve on HA. Using both a biomedical and multidimensional model of HA, we found that the multidimensional of HA was more inclusive and flexible and may be more useful for targeting public health interventions. Future research may be required to evaluate and monitor the predictive value of MMHA and BMHA in the older adult population in South Africa.

## Figures and Tables

**Table 1:** Sample and healthy ageing characteristics among older adults (≥50 years) in South Africa, 2011–2012.

Variable	Sample	Healthy Ageing Components					Healthy Ageing
		No Major Diseases	No Disability	Good Mental Health	Social Engagement	High Well-Being	
	N (%) or Med (IQR)	%	%	%	%	%	%
Sociodemographic factors							
All	3734	73.3	87.1	62.3	73.0	64.0	36.6
Age in years: Median (interquartile range)	59 (54–68)	59 (55–69)	59 (54–68)	59 (54–70)	59 (55–73)	59 (54–71)	58 (53–65)
Sex							
Female	2271 (56.8)	70.2	84.4	57.8	70.3	62.7	33.8
Male	1446 (43.2)	77.1	90.7	68.4	76.6	65.7	40.5
Population group							
Black African	2233 (60.3)	72.4	84.7	55.2	67.9	58.3	30.3
White	296 (8.0)	80.1	95.1	79.2	84.4	85.6	57.5
Coloured	741 (20.0)	73.0	91.1	78.7	86.5	66.0	43.1
Indian/Asian	433 (11.7)	56.1	84.6	71.6	83.2	60.2	29.7
Residential status							
Rural	1321 (38.7)	73.4	85.3	58.6	69.5	59.0	31.5
Urban	2413 (61.3)	73.2	88.2	64.5	75.1	67.0	39.8
Health risk behaviour							
Daily tobacco use	767 (18.7)	73.3	85.9	61.5	75.8	51.7	29.1
Problem drinking	558 (17.9)	78.6	89.7	68.0	76.3	64.8	40.0
Physical activity							
Low	1989 (51.5)	72.9	84.0	62.8	68.5	64.1	37.8
Moderate	402 (11.4)	72.4	90.1	64.7	81.4	64.9	39.3
High	1208 (37.1)	74.3	91.1	59.7	77.4	63.3	33.7
Sedentary behaviour	521 (15.6)	72.0	78.2	59.5	62.3	54.1	30.8
Vegetables (<once/day)	1564 (42.2)	75.4	85.7	60.9	70.6	60.3	36.7
Fruits (<once/day)	1705 (44.9)	75.3	86.0	59.4	70.6	58.5	35.4
Body weight perception							
Normal	2700 (71.1)	75.5	87.9	64.6	73.6	70.1	40.1
Underweight	403 (11.0)	70.3	81.5	46.6	63.5	36.2	20.8
Overweight	588 (17.9)	65.5	87.5	62.3	78.0	58.7	32.7

**Table 2:** Healthy ageing by biomedical and multidimensional models.

Successful Ageing Model and Components	Age Group			Biomedical Model	Multidimensional Model
	50–64	65–74	75+	50+	50+
	%	%	%	%	%
Biomedical model					
No disease: stroke, heart attack or angina, heart disease, diabetes, heart failure, rheumatic heart disease and psychological distress	52.6	48.0	36.7	49.7	
No disability (0 ADL)	89.9	86.8	71.6	87.1	
No cognitive impairment	94.6	89.4	73.6	91.0	
High physical functioning	78.8	69.2	50.8	73.4	
Social engagement	78.3	68.3	51.9	73.0	
Total	44.8	35.6	22.1	40.3	
Multidimensional model					
No disease: stroke, heart attack or angina, heart disease, diabetes, heart failure and rheumatic heart disease	74.5	72.2	68.3		73.3
No disability (0 ADL)	89.9	86.8	71.6		87.1
Good mental health	65.2	60.0	50.2		62.3
Social engagement	78.3	68.3	51.9		73.0
High well-being	67.2	60.9	51.6		64.0
Total	40.1	33.3	21.6		36.6

**Table 3:** Associations with healthy ageing.

	Multi-Dimensional Concept		Biomedical Concept
Variable	Crude Odds Ratio (95% CI)	Adjusted Odds Ratio (95% CI)	Adjusted Odds Ratio (95% CI)
Sociodemographic Factors			
Age in years	0.97 (0.95, 0.98) ***	0.96 (0.94, 0.97) ***	0.95 (0.94, 0.97) ***
Sex			
Female	1 (Reference)	1 (Reference)	1 (Reference)
Male	1.34 (1.04, 1.71) *	1.33 (1.03, 1.72) *	1.44 (1.13, 1.83) **
Population group			
Black African	1 (Reference)	1 (Reference)	1 (Reference)
White	3.10 (2.16, 4.47) ***	3.46 (2.29, 5.22) ***	3.02 (1.98, 4.60) ***
Coloured	1.74 (1.35, 2.25) ***	1.82 (1.34, 2.47) ***	2.02 (1.48, 2.74) ***
Indian/Asian	0.97 (0.63, 1.49)	0.90 (0.55, 1.46)	0.83 (0.49, 1.40)
Residential status			
Rural	1 (Reference)	1 (Reference)	1 (Reference)
Urban	1.44 (1.10, 1.88) **	1.05 (0.78, 1.41)	1.12 (0.85, 1.48)
Health risk behaviour			
Daily tobacco use	0.67 (0.51, 0.87) **	0.56 (0.42, 0.74) ***	0.68 (0.53, 0.88) **
Problem drinking	1.18 (0.84, 1.66)	---	---
Physical activity		---	---
Low	1 (Reference)		
Moderate	1.07 (0.72, 1.58)		
High	0.84 (0.65, 1.07)		
Sedentary behaviour	0.73 (0.48, 1.11)	---	---
Vegetables (<once/day)	1.00 (0.77, 1.30)	---	---
Fruits (<once/day)	0.92 (0.71, 1.21)	---	---
Body weight perception			
Normal	1 (Reference)	1 (Reference)	1 (Reference)
Underweight	0.39 (0.28, 0.54) ***	0.48 (0.34, 0.66) ***	0.64 (0.48, 0.85) **
Overweight	0.72 (0.49, 1.08)	0.53 (0.34, 0.81) **	0.68 (0.45, 1.03)

CI = Confidence Interval; *** *p* < 0.001; ** *p* < 0.01; * *p* < 0.05.

## Data Availability

The SANHANES-I data are available at http://www.hsrc.ac.za/en/research-data/ (accessed on 2 October 2019).
